# Symmetrical peripheral gangrene: A rare clinical entity

**DOI:** 10.1002/ccr3.3300

**Published:** 2020-09-12

**Authors:** Kudzai T. Macheka, Tasimbanashe Masamha, Hopewell Mungani, Linda Kumirayi

**Affiliations:** ^1^ Department of Surgery College of Health Sciences Parirenyatwa Hospital Harare Zimbabwe

**Keywords:** disseminated intravascular coagulation, purpura fulminans, septicemia, symmetrical peripheral gangrene

## Abstract

Symmetrical peripheral gangrene (SPG) may present initially with septicemia. Prompt identification and management of the underlying cause of SPG is central for the best outcome. Good management incorporates delaying early surgical intervention, suitable antibiotic therapy, judicious debridement, and skin grafting.

## Graphical abstract

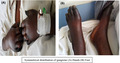

## 1 INTRODUCTION

Symmetrical peripheral gangrene (SPG) is an uncommon, potentially devastating clinical entity which may be associated with septicemia. A high index of suspicion, correct management including adequate resuscitation beginning at first point of contact improves the outcome of patients as SPG is associated with a high mortality rate (up to 35%).

Symmetrical peripheral gangrene also known as purpura fulminans is a rare clinical syndrome characterized by symmetrical gangrene of two or more extremities without obstruction of large vessels or vasculitis.^1^ This condition frequently affects the fingers and toes with the nose, ears, and genitalia being rarely affected.^1^ SPG was first described by Hutchinson in 1891 as gangrene of the distal extremities occurring with a symmetrical distribution.^2^ Several case reports with a few small series of patients with this condition have been reported in literature.^3^, ^4^ The etiological spectrum of SPG is wide, and it is important to identify and treat the underlying condition.

## 2 CASE REPORT

A 75‐year‐old female patient presented to tertiary referral academic hospital, Harare Central Hospital, from a provincial hospital with a 2 week history of pain and darkening of all her fingers and toes. A month prior to visiting hospital she developed a constant rest pain in her hands and feet. The pain was exacerbated by manual work and was not relieved by analgesia. She highlighted intermittent episodes of severe pain in her hands and feet occurring over the last 1 year. Her fingers and toes became dusky in appearance, and this was associated with blistering of the fingers and toes. The patient highlighted sensory loss in her fingers and toes. There was no history of intermittent claudication. The patient did not drink alcohol or smoke, and she had no known chronic illnesses. There was no family history of diabetes mellitus, hypertension, heart disease, dyslipidemias, and connective tissue disorders. The patient did not admit to a history of eating mouldy grain or bread. In the review of systems, she did not have any history of chest pain or palpitations. The patient did not have any history of headaches, seizures, or hallucinations. The past medical history was insignificant. She was negative for human immunodeficiency virus (HIV) and was not on any medications.

**FIGURE 1:**
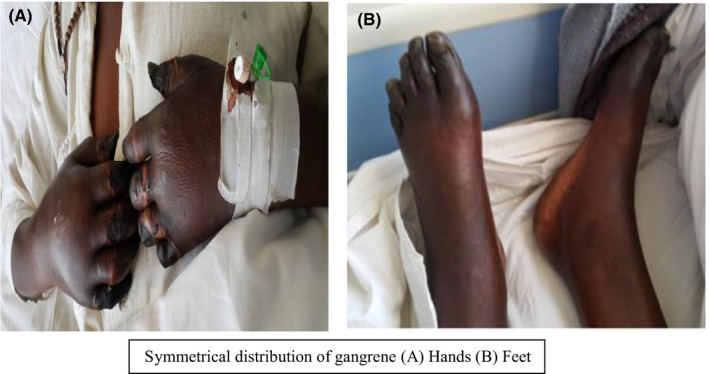
Symmetrical distribution of gangrene (A) Hands (B) Feet

Examination of the patient at the referral center revealed she was fully conscious, and not in any apparent distress. She had a normal pulse rate of 90 beats per minute, with a normal blood pressure (systolic reading of 128 mm Hg and diastolic of 59 mm Hg). She had a pyrexia of 37.8°C with no lymphadenopathy. There were no Janeway lesions or Xanthoma on examination. The cardiac, respiratory, and abdominal examinations were normal. On examination of the hands, the patient had dry gangrene of all the fingers of the hands which had demarcated. She had very good peripheral pulses in the upper limbs. In the lower limbs, she had dusky hyperpigmented toes. All the peripheral pulses in the lower limb were present and full volume. See Figure 1

The complete blood count on admission showed a raised white cell count of 16.1 × 10^9^/L, with a low hemoglobin of 11.6 g/dL. Her renal function and lipid profile were normal. A random blood sugar done on admission was within normal limits. A chest x‐ray and electrocardiogram (ECG) were done and were normal. An echocardiogram (Echo) was done as part of her workup, and she had a structurally normal heart with no thrombi and a good ejection fraction of 67%.

**FIGURE 2:**
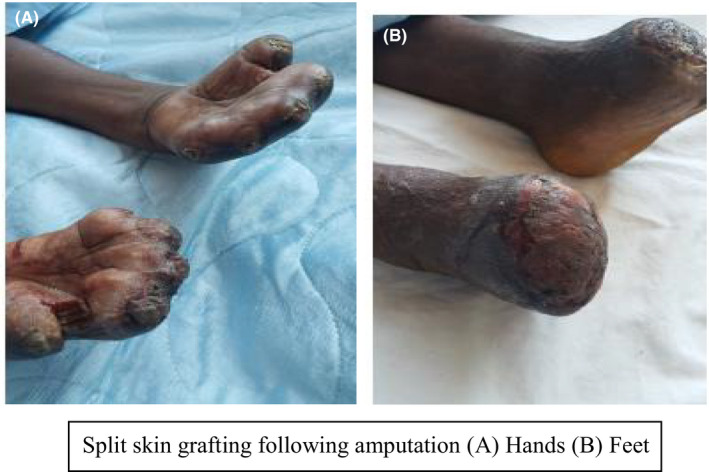
Split skin grafting following amputation (A) Hands (B) Feet

Fluid resuscitation was commenced with intravenous crystalloid solution and empiric antibiotics therapy with her pyrexia settling on the third day after admission. Her toes demarcated as dry gangrene by day 3 postadmission. The patient was consented for amputation of the gangrenous parts of her hands and feet. Amputation of the digits of her fingers was done with transmetartasal amputation of her feet done bilaterally. The surgery was uneventful. Two weeks after the initial surgery, the patient had split skin grafting to cover the bare amputation sites with excellent uptake of the grafts. See Figure 2. Pathological evaluation of amputated tissue did not report any vasculitis. There were microthrombi in the small vessels with sparing of the large caliber vessels.

She was discharged home 5 days after split skin grafting. The patient underwent physiotherapy and occupational therapy sessions at her local hospital to assist with rehabilitation and mobilization. On review 1 month later, all her wounds had healed and the patient was well with no complaints.

## 3 DISCUSSION

Symmetrical peripheral gangrene (SPG) is an uncommon condition associated with symmetrical ischemia and gangrene of the distal extremities. It can occur at any age and may affect either sex. SPG should be managed aggressively as it is associated with high morbidity and mortality. Most patients end up with amputation of the affected limbs. It is important to have a high index of suspicion and identify the underlying cause early as this is essential in preserving life and limb before irreversible ischemia and gangrene set in. SPG affects 2 or more extremities and is not associated with obstruction or vasculitis of large vessels.^1^

Symmetrical peripheral gangrene is associated with a wide spectrum of infective and noninfective etiological causes. Infection with resultant sepsis plays a major role in the etiology of SPG. Sepsis from bacterial infections like Pneumococcus, Neisseria meningitides, *Staphylococcus aureus*, and *S pyogenes* among other infections have been noted in patients with SPG.^5^ Rare infections which may cause SPG include severe infection from Plasmodium falciparum malaria.^6^ Noninfective causes of SPG include and are not limited to malignancy, hypovolemic shock, myeloproliferative disorders, vasospastic conditions, connective tissue disorders like systemic lupus erythematosus (SLE) and antiphospholipid antibody syndrome among other causes.^1^, ^5^, ^7^ Drugs like noradrenaline, adrenaline, and dopamine have been documented as causative agents in some patients with SPG.^1^, ^8^ Identifying and treating the underlying cause helps to arrest and prevent further progression in SPG.^5^

The exact pathogenesis of how SPG occurs is not well understood. The underlying mechanism includes a low flow state with disseminated intravascular coagulation (DIC).^1^, ^4^ Molos et al^9^ noted DIC as an important underlying factor in 85% of patients who develop SPG. Davis et al^3^ reported similar findings in his cohort of 12 patients with SPG. SPG manifests unfavorably in patients with immunosuppression, asplenism, hypothermia, diabetes mellitus, and renal failure.^10^ SPG should be suspected when a patient presents with marked coldness, pain in the distal extremities, cyanosis, and pallor. Early recognition helps to arrest progression of ischemic changes before overt gangrene occurs. Ischemic changes usually begin in the peripheries and extend proximally. Ischemic changes are not preceded by peripheral vascular occlusive disease. The distal pulses in the large vessels are intact.^5^ DIC and an associated low flow state may result in occlusion of the microcirculation in the affected limbs with resultant ischemia and gangrene. Pathological evaluation of amputated specimens has shown thrombi which are mainly found in small vessels sparing the large vessels. There is no associated vasculitis as well.^11^ This is important as focus should not be on the distal ischemic changes only without addressing the underlying disease process.

Despite a wide array of etiological causes for SPG, it is not uncommon to fail to identify an underlying cause. This may be particularly challenging in a low resource setting where discriminatory investigations to identify the cause may be difficult to undertake. An important learning point in this case report is that lack of diagnostic investigations should not compromise urgent and timely management of SPG. Delays in managing patients with SPG may result in poor outcomes for patients. A limitation of this case report was the inability to do all the relevant investigations including a full infection screen. The patient was pyrexial with a raised white cell count suggesting an underlying infectious process as the cause. Laboratory tests like a blood culture, serum lactate levels, and D‐dimers to evaluate for possible underlying DIC could not be done. The various laboratory reagents and blood culture bottles were not available at the time in the hospital.

Symmetrical peripheral gangrene is a diagnosis of exclusion, and other causes of gangrene must be excluded. The aim is to address and treat the underlying cause. Some of the differentials for SPG include thomboangitis obliterans, thromboembolic gangrene, calciphylaxis, and vasculitic gangrene.^5^ These were unlikely causes in our patient as supported by her history and investigations. She was of sober habits with good cardiac function. On examination, she had symmetrical gangrene involving more than two distal extremities and all her peripheral pulses were present and full volume. Pathological examination of vessels sent for histology did not show any underlying vasculitic process. These findings were in keeping with SPG. Patients with Raynaud's phenomena may present with symptoms comparable to SPG. However, in Raynaud's phenomena, they rarely develop gangrene and have a history of failing to tolerate cold weather. Attacks of vasospasm of the fingers and toes in Raynaud's phenomena are frequent and triggered by stress and exposure to cold.^12^ Our patient did not admit to any of these symptoms. A potential cause of SPG could have been ergot poisoning; however, the patient did not highlight a history of ingesting mouldy grain or bread. She did not have neurological symptoms like convulsions and hallucinations to suggest it as a potential etiology.

Choice of investigations for patients with suspected SPG should be guided by clinical features and must be individualized to each patient. An infection screen is important to rule out sepsis and DIC. Blood investigations like a full blood count, a blood culture, and a peripheral smear are useful to assess for sepsis.^1^ Presence of schistocytes on peripheral smear may be an indicator of DIC.^5^ A peripheral smear will additionally help assess for malignancy and other causes of SPG like Plasmodium falciparum. Patients may be assessed for DIC by measuring the blood lactate levels, D‐dimer assay, and assessing for fibrin degradation products. Other useful tests when indicated include blood tests for connective tissue disorders like the antinuclear antigen test (ANA) and antiphospholipid antibodies (APLA).^4^, ^5^ Imaging investigations like a Doppler USS may be done but do not alter the course of management. Peripheral Doppler examination will highlight absence of involvement of the large peripheral vessels.^5^

Patient management involves adequate resuscitation with use of broad spectrum antibiotic cover particularly in patients with sepsis and DIC. Patients who develop a bleeding diathesis should have it corrected with replacement of depleted clotting factors.^1^ Prostacyclin (epoprostenol) and tissue plasminogen infusion have been used with some success in managing patients with SPG. Plasmapheresis, leukapheresis, and sympathetic blockade may be beneficial but have a limited role in SPG.^1^, ^13^ Use of anticoagulants like heparin and aspirin is controversial. Johansen and Hansen reported that heparin, aspirin, and streptokinase were not effective in preventing progression of gangrene when used in patients with SPG. The addition of oral corticosteroids did not confer any treatment benefit as well.^14^

Symmetrical peripheral gangrene may have a mortality rate as high as 35% with most patients inevitably requiring an amputation.^4^, ^9^ Initially, a nonsurgical approach is appropriate with emphasis on stabilizing the patient and treating the underlying condition. This allows the gangrene to demarcate before an amputation is done.

## 4 CONCLUSION

Symmetrical peripheral gangrene is a rare condition whose therapeutic management is anecdotal and based on case reports and a few case series. No treatment has been noted to be universally effective in SPG. Treatment must be individualized to the patient. The most important factors when managing patients with SPG is to address the underlying disease process so as to get the best possible outcome for the patient. Focus should never be on the ischemic and gangrenous process in the peripheral limbs alone but addressing the underlying pathophysiologic disease process and managing the patient holistically to get the best outcome.

## CONFLICT OF INTEREST

None.

## AUTHOR CONTRIBUTIONS

KM and TM: involved in the case report design, subject research, consent, editing, and writing. HM and LK: involved in the subject research, editing, and writing.

## ETHICAL APPROVAL

Ethical approval was exempted by our institution.

## CONSENT

Written informed consent was obtained from the patient for publication.
